# A Review of Controversial Issues in the Management of Head and Neck Cancer: A Swiss Multidisciplinary and Multi-Institutional Patterns of Care Study—Part 2 (Radiation Oncology)

**DOI:** 10.3389/fonc.2019.01126

**Published:** 2019-10-24

**Authors:** Olgun Elicin, Paul Martin Putora, Marco Siano, Martina A. Broglie, Christian Simon, Daniel Zwahlen, Gerhard F. Huber, Giorgio Ballerini, Lorenza Beffa, Roland Giger, Sacha Rothschild, Sandro V. Negri, Pavel Dulguerov, Guido Henke

**Affiliations:** ^1^Department of Radiation Oncology, Inselspital, Bern University Hospital, University of Bern, Bern, Switzerland; ^2^Department of Radiation Oncology, Cantonal Hospital St. Gallen, St. Gallen, Switzerland; ^3^Department of Medical Oncology, Cantonal Hospital St. Gallen, St. Gallen, Switzerland; ^4^Department of Medical Oncology, Hôpital Riviera-Chablais, Vevey, Switzerland; ^5^Department of Otorhinolaryngology, Head and Neck Surgery, Cantonal Hospital St. Gallen, St. Gallen, Switzerland; ^6^Department of Otorhinolaryngology, Head and Neck Surgery, University Hospital Zurich, Zurich, Switzerland; ^7^Department of Otorhinolaryngology, Head and Neck Surgery, University Hospital of Lausanne, Lausanne, Switzerland; ^8^Department of Radiation Oncology, Cantonal Hospital Graubünden, Chur, Switzerland; ^9^Department of Radiation Oncology, Cantonal Hospital of Winterthur, Winterthur, Switzerland; ^10^Department of Radiation Oncology, Clinica Luganese SA, Lugano, Switzerland; ^11^Department of Radiation Oncology, Cantonal Hospital Lucerne, Lucerne, Switzerland; ^12^Department of Otorhinolaryngology, Head and Neck Surgery, Inselspital, Bern University Hospital, Bern, Switzerland; ^13^Department of Medical Oncology, University Hospital of Basel, Basel, Switzerland; ^14^Department of Otorhinolaryngology, Lindenhofspital, Bern, Switzerland; ^15^Department of Otorhinolaryngology, Head and Neck Surgery, Geneva University Hospital, Geneva, Switzerland

**Keywords:** consensus, head and neck cancer, patterns of care, practice patterns, survey

## Abstract

**Background:** The Head and Neck Cancer Working Group of Swiss Group for Clinical Cancer Research (SAKK) has investigated the level of consensus (LOC) and discrepancy in everyday practice of diagnosis and treatment in head and neck cancer.

**Materials and Methods:** An online survey was iteratively generated with 10 Swiss university and teaching hospitals. LOC below 50% was defined as no agreement, while higher LOC were arbitrarily categorized as low (51–74%), moderate (75–84%), and high (≥85%).

**Results:** Any LOC was achieved in 62% of topics (*n* = 60). High, moderate, and low LOC were found in 18, 20, and 23%, respectively. Regarding Head and Neck Surgery, Radiation Oncology, Medical Oncology, and biomarkers, LOC was achieved in 50, 57, 83, and 43%, respectively.

**Conclusions:** Consensus on clinical topics is rather low for surgeons and radiation oncologists. The questions discussed might highlight discrepancies, stimulate standardization of practice, and prioritize topics for future clinical research.

## Introduction

This is the second part of the article “A Review of Controversial Issues in the Management of Head and Neck Cancer: A Swiss Multidisciplinary and Multi-Institutional Patterns of Care Study,” providing the results for the items concerning radiation oncology discipline, each followed by a short discussion if deemed relevant.

The details of the methodology is presented in the first part of this series.

## Results and Discussion

### Radiation Oncology

#### Definition and Compartmentalization of Target Volumes

➢ *Omitting the elective treatment of the contralateral neck is safe in well-lateralized primaries of the tonsil: moderate LOC (80%)*.

For a cT2 carcinoma of the tonsil, the uninvolved contralateral neck is omitted if the tumor is well lateralized and with <10 mm of the superficial mucosa of soft palate and/or base of tongue in 8/10 centers. The remaining two centers always perform bilateral treatment.

Although no prospective randomized trial was performed to exclusively answer this question, there is mounting evidence to support the safety of ipsilateral treatment of well-lateralized OPSCC. As endorsed by the American College of Radiologists, treatment can be limited to the ipsilateral side in tonsil primaries with a N0-1 nodal stage when the primary exhibits <1 cm invasion into the soft palate or base of tongue (1). Other retrospective series also showed excellent results with N2b or unilateral N3 cases (2–4) and in other oropharyngeal (2, 5) as well as oral cavity subsites (6). However, no prospective randomized trial results for this question are available. In the recently updated international consensus guidelines, this issue is still regarded as controversial, and caution is advised especially for nodal stages above N2a (7).

➢ *Compartmentalization of the tumor bed and the levels of the nodal basin for post-operative radiotherapy in terms of dose and volume: no consensus*.

In 3/10 centers, the post-operative primary tumor bed is not included in the target volumes, if the indication for adjuvant radiotherapy arises only due to nodal factors after neck dissection. The remaining 7 centers do not separate the tumor bed and the dissected nodal levels.

Similarly, regarding the elective/low risk volumes in the post-operative setting, in 5/10 centers the whole post-operative neck is considered as an inseparable target compartment. In the other half of the centers, the levels are thought of separable compartments, and, in eligible cases based on the nodal distribution pattern reported by the pathology, radiotherapy to a portion/level of the post-operative neck is omitted.

The selection of radiotherapy target volumes is strongly influenced by tradition. More than a decade ago, the landmark EORTC 22931 and RTOG 9501 trials defined the major and minor risk factors for the indications of post-operative CRT and radiotherapy, respectively. However, the question of the necessity of such an “all or nothing” approach concerning different parts of the target volume(s) remains unanswered. Surely, one of the arguments for irradiating the primary tumor bed in case of multiple nodes with or without ECE has been the general loco-regional recurrence risk and difficulties to irradiate the primary tumor recurrences after previous nodal irradiation, especially in the past due to technical limitations. Nevertheless, from a purely medical and not a technical perspective, it is not clear, why the post-operative primary tumor bed should be irradiated due to multiple nodal positivity and/or ECE, whereas the same patient and tumor bed would not receive any radiation if the neck would have been pN0-1. Similarly, there is no data indicating perineural extension as a risk factor for nodal recurrence.

Concerning the post-operative nodal target volume, half of the radiation oncologists still treat the entire surgical bed covering both the primary tumor bed and the operated neck (at least the involved side). On the other hand concerning the post-operative primary tumor target volume, most oncologists still treat the entire surgical bed at least within a low risk volume irrespective of risk factors specifically related to the primary tumor or the neck (8). Nevertheless, the recently demonstrated long-term results of a prospective phase II study supports the safety of this compartmentalization approach (9). On the contrary, data indicating the risk of compartmentalization approaches also exist (10). However, such retrospective studies reporting unusually high recurrence rates should be critically interpreted in the lack of description of surgical techniques and radiotherapy approach especially in terms of online and offline image guidance protocols within the frame of the limited volume approach.

➢ *Adaptation of the dose or target volumes (except for the replacement of anatomical barriers) after induction chemotherapy is not preferred: moderate LOC (80%)*.

After an induction chemotherapy, 8/10 centers would not adapt the dose or target volume (except for anatomical changes) regardless of a partial or complete response. In one center clinical target volume (CTV) would be adapted based on tumor shrinkage. In another center, both dose and volume would be de-escalated based on response.

For radiotherapy planning after induction chemotherapy radiotherapy, Salama et al. (11) recommended the irradiation of pre-induction volumes with full dose even in case of a clinical complete response while taking the volumetric changes in anatomical structures and barriers into consideration. Despite of that, there is a substantial heterogeneity in target volume definition concepts among different institutions (12, 13). Although not part of the main scientific question and primary endpoint, the target volumes and prescribed doses after a clinical response to induction chemotherapy were adapted in some contemporary prospective clinical trials (13, 14). In a recently published phase III randomized trial the non-adapted and adapted volume approaches after induction chemotherapy for nasopharyngeal cancer were compared (15). The investigators did not report any inferior oncologic outcome with the adapted strategy. However, volume reduction did not result in a substantial reduction of toxicity or improvement in quality of life except for a few among the many investigated domains. It is also worth to note, that this study was underpowered to detect a non-inferiority in oncologic outcome in this regard. Moreover, there are quantitative analyses indicating that it is unsafe to adapt the high-risk volume based on the shrinkage of the macroscopically visible tumor in radiological imaging after a non-definitive treatment (16).

➢ *Definition of treatment volumes for the treatment of CUP: no consensus*.

No consensus was reached concerning the treatment volumes in CUP situation. Treatment volumes of a CUP always contain bilateral neck and potential mucosal sites (4/10); only the involved side(s) of the neck (3/10); and involved side(s) plus corresponding mucosal sites only in case of human papillomavirus (HPV) or Epstein-Barr Virus (EBV) positivity (2/10). One center always treats the mucosal sites but only with the involved side(s) of the neck.

The literature about the optimal management of CUP is conflicting. There is no convincing data supporting the elective irradiation of the contralateral uninvolved neck in the modern series (17–19), whereas the reports indicating the superiority of bilateral irradiation are outdated in terms of radiotherapy and imaging modalities (20). Some facts are worth considering for the selection of the optimal strategy (21–25): (1) The risk of nodal recurrence and distant metastases is at least twice higher than the subsequent appearance of a mucosal primary tumor (≤ 10%). (2) The emergence rates of mucosal primary tumors after unilateral neck irradiation are similar to the risk of occurrence of metachronous second primary tumors in patients cured of a known head and neck SCC primary. (3) Survival rates are not related to the appearance of the primary tumor (21, 22, 26). Last but not least, doubling the target volume by means of bilateral irradiation substantially contributes to the toxicity burden, which would outweigh any marginal oncological benefit, which rather seems non-existent (18, 19).

➢ *Use of an isotropic margin and respecting the anatomical barriers is the preferred method to generate high-risk CTVs around the gross tumor volume (GTV): low LOC (60%)*.

When contouring the high risk CTV around the primary tumor, 3/10 centers use the predefined anatomical subsites defined by Eisbruch et al. (27). One center treats these sites with 60 Gy by using an intermediate risk volume. The rest of the centers only use an anatomical isotropic margin and crop this volume from the anatomical barriers as suggested by Caudell et al. (28), who also reported a non-inferior outcome with the geometric extension approach compared to treatments with predefined anatomical subsites.

The survey was completed before the recent publication of the international consensus guidelines for the delineation of the primary tumor CTV by Grégoire et al. (29), in which the isotropic geometric expansion concept was also endorsed. These guidelines recommend the use of 5 and 10 mm around the GTV for high-risk and prophylactic CTVs, respectively. Nevertheless, these volumes shall be manually cropped by taking the anatomical barriers into account. The exceptions to this rule were defined for early stage glottic and locally-advanced stage hypopharyngeal primaries. For the former, prophylactic volumes were deemed unnecessary, whereas for the latter, a 15 mm margin in the cranio-caudal direction was suggested.

➢ *A restricted use of intermediate-risk dose only in the levels with ECE is preferred: high LOC (90%)*.

In case of pathologically-confirmed ECE, only the involved levels are treated with an intermediate dose of 60–66 Gy in 9/10 centers. The rest of the neck is treated with an elective/low-risk dose. In one center, all involved levels are treated with 64 Gy irrespective of ECE, and the uninvolved levels are treated with a lower dose, since systematical anatomical marking of the lymph node levels on the surgical specimen is not performed sufficiently.

Traditionally, some head and neck cancer oncologists were concerned about the intraoperative spillage of the tumor cells, in case of ECE and/or positive resection margins. However, even in the twin landmark RTOG (9501) (30) and EORTC (22931) (31) trials, only the high risk areas were boosted up to 60–66 Gy. In the current international consensus guidelines for the delineation of nodal target volumes, a compartmentalized approach is recommended. It is worth to note, that the evidence level supporting the inclusion of non-involved postoperative levels into the prophylactic volumes even in the N+ neck is low, and this approach is rather based on tradition (8, 27). Nevertheless, it seems, that it is not always possible for the radiation oncologists of these 9 centers to compartmentalize the intermediate-risk volume, since only 4 centers systematically mark the lymph node levels on the surgical specimen before sending them to the pathology.

➢ *Use of tailored planning target volumes (PTV) for different anatomical subsites: no consensus*.

In some anatomical subsites (e.g., larynx, tongue, soft palate), 4/10 centers use additional geometric margins concept to compensate for possible organ movement. In one of these centers, an anisotropic margin for larynx and soft palate primaries are used. For the remaining 6 centers, such an internal target volume concept is not used based on subsite. On the other hand, the policy of these centers is to re-plan and adapt the margins according to movement based on daily imaging, if considered necessary.

The conventional fields in the 2D radiotherapy era encompassed the target volumes with enough margins to compensate for movement. As an example, the larynx is known to move up to 20–25 mm craniocaudally (32, 33). Despite of that, the traditional 2D fields did not require further enlargement due to the technical features of 2D-conventional radiotherapy (32). However, the sharp dose fall-off profile of intensity-modulated radiotherapy (IMRT) to spare sensitive tissues allows less tolerance for target volume delineation errors and marginal misses. Studies performed with volumetric imaging and dynamic MRI demonstrated the necessity of extra margins of 5 mm to every, and 6–7 mm to cranial direction for the primaries of soft palate, larynx, and hypopharynx (34, 35). Recently published data by Bruijnen et al. (36) demonstrate considerably shorter ranges of intrafractional tumor motion <3 mm (95th percentile—excluding swallowing) with a decreasing order from laryngeal to oropharyngeal and nasopharyngeal primaries, respectively. However, in addition to intrafractional, the interfractional positional differences of soft palate, uvula, larynx, and tongue; moreover, the elastic changes in the relationship of different subvolumes of PTV [e.g., primary tumor and involved lymph node(s)] are more difficult to quantify and to tackle with. Unacceptable variations seen with daily imaging should lead to adaptive re-planning as quickly as possible. As a less systematically reported issue, swallowing frequency, and positional changes in the pharyngo-laryngeal anatomy during the treatment may be associated with changing treatment anxiety, consistency of saliva, and increasing mucositis throughout the course of treatment.

➢ *Definition of high- and low-risk volumes for laryngeal primaries: no consensus*.

Laryngeal primaries are treated by including the whole larynx in the high-risk volume in 2/10 centers. Five centers prefer to treat the primary tumor with a predefined margin. In the rest of the centers, the larynx (in one center the involved hemilarynx) is considered as a compartment which shall be treated with an elective dose. The primary tumor is treated to a high dose with a predefined margin.

The 3D volume definition for laryngeal primaries was just a translation of traditional 2D fields to the 3D era. This resulted in the continuation of treating the whole larynx within the high-risk volume receiving the highest dose, even for early stage tumors without infiltration to cartilaginous structures, contralateral extension, etc. This concept is still being used in some centers. At the other end of the spectrum, hemilarynx (37, 38), even single vocal cord irradiation (39) techniques were developed for early stage laryngeal primaries, yielding excellent results. For locally advanced laryngeal primaries, the inclusion of the whole larynx into the prophylactic target volumes is not recommended anymore by current consensus guidelines (29).

#### Dose and Fractionation Concepts

➢ *The use of simultaneous integrated boost (SIB) is the preferred boost technique: moderate LOC (80%)*.

Centers were asked to provide information about the boost techniques and dose/fractionation regimens for target volumes (Table 1). Simultaneous integrated boost (SIB) and sequential boost (SEQ) techniques are used in 8 and 2 centers, respectively.

**Table 1:** Dose-fractionation schedules for definitive (chemo)radiotherapy.

**Center**	**1**	**2**	**3**	**4**	**5**	**6**	**7**	**8**	**9**	**10**
High risk dose (Gy)/fractions	69.96/33	72/36	70/35	66/30	69.63/33	70/35	69.3/33	70–76/35-38^*^	69.63/33^#^	70/33
Intermediate risk dose (Gy)/fractions	59.4/33	66/33	66/33	60/30	66/33	60/35	56.1/33	64/32	60/33	59.4/33
Low risk dose (Gy)/fractions	52.8/33	54/30	50/25	54/30	56/33	54/35	52.8/33	50–54/25-27^*^	54/33	52.8/33
Boost technique	SIB	SIB	SEQ	SIB	SIB	SIB	SIB	SEQ	SIB	SIB

*SEQ, sequential boost; SIB, simultaneous integrated boost*.

* *Higher dose in case of no concomitant systemic therapy*.

# *70 Gy in 35 fractions for “large” tumors*.

IMRT with inverse planning allows SIB to multiple target volumes during the course of radiotherapy by means of a dose painting approach. The beams used to deliver the planned dose to the high-risk volume are exploited for the dose application to the encircling low-risk volume(s). In contrast to the traditional sequential shrinking field/volume approach, SIB enables the generation of single-phase plans with the possibility of a more flexible plan optimization process. This allows an advantage over SEQ in terms of better control of dose around the high risk PTV and reducing the unwanted high dose areas within. Although there are countless retrospective and prospective studies, in which patients were treated with SIB, no prospective randomized trial compared both technical modalities until recently. Lertbutsayanukul et al. (40) conducted a phase III randomized trial with the primary endpoint of acute and late toxicities during and after SIB vs. SEQ for the treatment of nasopharyngeal cancer. This study with a superiority design did not show any statistically or clinically significant difference in toxicity or oncologic endpoints. In theory, similar studies including other four major HNSCC subsites are needed. However, the toxicity results reported by Lertbutsaanukul et al. can be extrapolated to other subsites, considering the fact, that the treatment of nasopharyngeal cancer involves the largest and most complex target volume and organs at risk in the head and neck area.

➢ *Hypofractionation for the treatment of early stage glottic larynx cancer: no consensus*.

For early stage glottic larynx cancer, 4/10 centers perform hypofractionated radiotherapy (≥2.25 Gy per fraction).

There is mounting evidence supporting the shortened treatment time in the treatment of stage I-II glottic larynx cancer for increased tumor control (41). Reports on large series from cancer registries (42, 43), prospective clinical databases (44), meta-analyses (41), and prospective randomized trials (45–47) demonstrated favorable results with altered fractionation either by means of hypofractionation and/or acceleration. The possible effect of hypofractionation is probably based on its treatment-accelerating effect, rather than the exploitation of the β value (44, 45, 48, 49). As reported so far, long-term toxicity is not a major point of concern with accelerated or moderately-hypofractionated irradiation (46, 47, 50), which is in line with the biological rationale regarding the time factor (49). It can be safely applied and may be preferred due to its benefits in terms of costs, logistics, and patient comfort. Hypothetically, the therapeutic window may also be widened with the use of contemporary treatment techniques (39). In this regard, impressive clinical results of a prospective study using SBRT (58.08 Gy in 16 fractions) with the primary endpoint of voice quality deserves attention (39): 2 years local control and overall survival of 100 and 90%, respectively, without any grade 3 or above toxicity. When compared with a historical control group, which was treated to the whole larynx (66 Gy in 33 fractions), single vocal cord irradiation yielded less grade ≥2 acute toxicity (17 vs. 66%, *p* < 0.01) and lower voice handicap index scores in almost all follow-up visits performed in regular short intervals until 18th month (*p* < 0.01). In contrast, a recently published phase I trial with extremely hypofractionated radiotherapy using robotic SBRT yielded inferior local control and not necessarily less toxicity compared to the literature (51). This was possibly because of the irregular laryngeal motions occurring during a protracted dose delivery and the lack of the current robotic SBRT unit's capability to handle them.

➢ *Altered fractionation is preferred in case of radiotherapy without concomitant systemic agents: moderate LOC (70%)*.

Altered fractionation is used in 7/10 centers. In the corresponding question, altered fractionation was defined as any treatment not fitting to the following arbitrary description in the questionnaire: single fraction/day throughout the whole treatment course with a fraction size between 1.8 and 2.2 Gy for the high-risk volume. The distribution among the altered fractionation regimens were as following: acceleration (six fractions per week or concomitant boost) in 6 centers, hyperfractionation in 3 centers (two centers use both strategies). Three centers combine systemic agents with hyperfractionation and/or acceleration.

Compared to normofractionated radiotherapy, the survival and loco-regional control benefit of altered fractionation is proven, particularly in the form of hyperfractionation in the definitive radiotherapy setting without concomitant systemic treatment (52). However, this added benefit of altered fractionation wanes out with increasing age (53), most probably due to competing risks for death, such as comorbidities. Therefore, the role of altered fractionation may be questioned in the selected elderly and/or fragile patients who are deemed not to tolerate systemic treatment.

There are numerous combinations of systemic agents and altered fractionation schedules for the treatment of HNSCC (54). In summary, there seems to be no benefit of combining accelerated fractionation and concomitant chemotherapy. For example, the GORTEC 99-02 trial randomized 840 patients into three arms with the primary endpoint as loco-regional control. In one of the two arms with chemotherapy (carboplatin and 5-fluorouracil), patients received 70 Gy in 35 fractions over 7 weeks, and in the other arm 70 Gy in 40 fractions over 6 weeks (40 Gy in 20 fractions over 4 weeks followed by 30 Gy in 20 fractions over 2 weeks). At 7 years, the difference in outcome was statistically not significant among the arms. Acute mucositis and feeding tube requirement were higher with accelerated radiotherapy by means of concomitant boost and chemotherapy than normofractionated radiotherapy and chemotherapy. Late toxicities were comparable (55, 56). The RTOG 0129 randomized 743 patients into two arms, both with concomitant cisplatin: normofractionated radiotherapy (70 Gy in 35 fractions over 7 weeks with three cycles of cisplatin) versus accelerated radiotherapy by means of concomitant boost (36 Gy in 18 fractions over 3.5 weeks followed by 36 Gy in 24 fractions over 1.5 weeks with two cycles of cisplatin). At 8 years, no significant difference in overall survival (primary endpoint), any oncological endpoints, or acute and late toxicities was observed (57). The question left unanswered is whether there would be an added benefit of combining hyperfractionated radiotherapy and concomitant chemotherapy compared to conventionally fractionated radiotherapy and chemotherapy. The statistical models indicate a potential advantage in this regard (58), which needs to be confirmed by prospective randomized trials. Unfortunately, it is quite unlikely to witness any large-scale trials conducted to answer this question due to the lack of financial attractiveness for the industry. The EORTC 22962 trial would have been the ideal phase III study with four arms, comparing normofractionated radiotherapy (70 Gy in 35 fractions) with hyperfractionated radiotherapy (80.5 Gy in 70 fractions) in 7 weeks with or without cisplatin. Unfortunately, the trial terminated prematurely due to slow accrual after recruiting only 57 patients. The above-mentioned RTOG 0129 was designed with the MD Anderson combined boost schedule. It is unknown what would have happened if the hyperfractionated arm of the RTOG 9003 (59) was chosen instead of the accelerated regimen.

➢ *There is no standard in terms of dose prescription and plan normalization: no consensus*.

During the radiotherapy planning process, 5/9 centers use the median dose to PTV for dose prescription. Of those, only 2 centers normalize the plan according to a minimum dose coverage criterion (e.g., D_95%_ = 95% of the prescribed dose).

The authors of the ICRU 83 report (60) only suggested to prescribe on the median absorbed dose to the target volume (D_50%_), but without a strict restriction of the use other dose-volume prescription values. In practice, there is a large variety in internal clinic protocols and clinical trial protocols. As an example, in the modern EORTC trials for HNSCC (e.g., NCT02984410, NCT01880359), it is requested to prescribe the dose on D_50%_, and obtain a dose coverage of at least 95% of the prescribed dose to the 95% of the PTV, whereas normalization to D_95%_ instead of D_50%_ is demanded in the RTOG protocols (e.g., NCT01302834, NCT01953952, NCT00265941). It is likely, that no consensus will exist in the near future. Nevertheless, it is important to be aware of these differences to correctly implement the dose, fractionation, and incorporate new techniques used in clinical trials into routine practice.

#### Evaluation of the Treatment Response

➢ *Refer to* *Table 2* *for LOC for each post-treatment response evaluation modality for the neck*.

**Table 2:** Post-(chemo)radiotherapy response evaluation schemes for stage III-IV/B disease.

**Center (LOC)**	**1**	**2**	**3**	**4**	**5**	**6**	**7**	**8**	**9**	**10**
Physical examination (no)	X	X	X			X				
Morphologic imaging (moderate)	X	X	X	X	X	X		X		X
Metabolic imaging (moderate)	X	X	X	X	X	X	X	X		
Regular ultrasound ± FNA (moderate)				X	X	X	X	X	X	X
Time interval for the post-treatment imaging in weeks (high LOC around 12, but no LOC for a strict time frame)	8–12	6–8	12–16	10–12	4–12	8–12	12	12	6–12	10–12

*FNA, fine-needle aspiration; LOC, level of consensus*.

The participating centers were asked to provide their post-(chemo)radiotherapy response evaluation schedules, which are summarized in Table 2. Morphologic and metabolic imaging modalities are the most frequently (8/10 for each) used tools for the assessment of treatment response, whereas there is a prominent heterogeneity regarding the regular use of physical examination, ultrasound (± fine-needle aspiration) and the time interval to perform these imaging examinations. There is no center, in which no regular post-treatment response evaluation imaging is performed.

Although there is no international consensus about the post-(chemo)radiotherapy response evaluation tools and the optimal time interval, the highest level of evidence was generated by the PET/NECK Trial (61), which demonstrated the futility of the planned neck dissection approach after CRT. Despite of being a relatively expensive imaging modality on its own, ^18^FDG-PET/CT is indeed cost-effective (62) compared to planned neck dissection and yields similar outcome in terms of survival and quality of life (61).

For response evaluation, ^18^FDG-PET/CT is reported to have a higher accuracy in the detection of recurrent lesions when compared to CT and MRI (63). Its negative predictive value is very high, but the positive predictive value is suboptimal. In other words, ^18^FDG-PET/CT is an ideal modality to rule out residual disease after (chemo)radiotherapy. Recent studies demonstrated further increased accuracy with delayed image acquisition around 16 weeks after treatment with NPVs reaching 100% (64–66). On the other hand, the access to ^18^FDG-PET/CT in low-cost setting is not always warranted, and morphologic imaging alone with MRI or CT should be relied on. Another well-known issue is the delayed response in involved lymph nodes of HPV+ oropharyngeal tumors (67), which sometimes exceeds 24 weeks after the end of treatment. Such patients are under increased risk of undergoing unnecessary biopsies and salvage neck dissections. Nevertheless, that does not mean, that the suspicious findings which indicate an incomplete remission (regardless of HPV status) can be left to routine clinical observation without performing a timely pathology examination.

The rationale of a regular ultrasound ± fine-needle aspiration policy (regardless of clinical response) is not clear, especially if the above-mentioned imaging modalities are already planned.

#### Palliative Radiotherapy and Salvage Re-Irradiation

➢ *No particular preference exists for palliative radiotherapy regimens: no consensus*.

Among centers, there was a heterogeneity in palliative radiotherapy regimens. Three centers did not provide any preferred regimen. The most frequently mentioned regimen was the Australian Quad-Shot (4/10). Details are provided in Table 3.

**Table 3:** Dose-fractionation schedules for palliative radiotherapy.

**Center**	**1**	**2**	**3**	**4**	**5**	**6**	**7**	**8**	**9**	**10**
Preferred regimen (dose in Gy/fraction)	None	50/20	QS	None	QS	QS^*^ or 37.5/15^#^	40/16 or 45/18	42/13^*^ or 12/2^#^	QS^*^ or 25/5^#^	None
Number of fractions/week	NA	5	4	NA	4	4^*^ or 5^#^	5	5^*^ or 2^#^	4^*^ or 5^#^	NA

*NA, not available; QS, Quad-Shot, i.e., 3 cycles of (4 × 3.5–3.75 Gy BID in 2 days) each 4 weeks apart*.

*,# *The values with these signs under each column correspond to the preferred regimen and the number of fractions under the same column*.

There are various radiotherapy regimens for the palliative treatment of head and neck cancer (68). In the lack of evidence to back a particular dose-fractionation regimen, the following aspects of palliative radiotherapy concept should be considered. Shorter treatment time and hospital visits play an important role for patient comfort. Hypofractionation and split-course regimens are safe in palliative setting (69). However, previously applied doses and normal tissue reserves should be always taken into consideration when choosing the optimal dose and fractionation. The use of IMRT is recommended to further minimize treatment toxicity.

➢ *Hypofractionated stereotactic body radiotherapy (SBRT) is considered in re-irradiation setting with curative intent: low LOC (60%)*.
➢ *SBRT is considered for palliative irradiation: low LOC (60%)*.

SBRT is performed in (or via referral to another center) 6/10 centers with an indication for re-irradiation with a curative intent. In 6/10 centers (partially overlapping with the former) it is used for palliative treatments. In one center, it is also used to apply the boost dose following the elective course of radiotherapy. In 2/10 centers it is never used.

Various applications of SBRT in head and neck cancer are reported (its use in glottic larynx cancer is mentioned previously):

1. Prospective clinical trials investigated the role of SBRT in re-irradiation of unresectable recurrences. The dose fractionation schedules were extremely hypofractionated (70– 72). Although no head-to-head comparisons exist, the survival rates seem to be not inferior to normofractionated (73, 74) or hyperfractionted (75, 76) schedules, and the toxicity profiles look comparable with slightly being superior (77). The last phase II trial (*n* = 50) demonstrated 6% acute and 6% late grade 3 toxicity rates with 40–44 Gy in five fractions over 2 weeks (72). The same group also published the largest retrospective series so far (*n* = 291) (78). The results of this study indicate, that the SBRT is safe and effective. Nevertheless, due to higher risk for late toxicity, the laryngeal and hypopharyngeal primaries should be carefully selected (72, 78).
  IMRT appears to be a feasible alternative as well (77). Recently, the Multi-Institution ReIrradiation (MIRI) Collaborative defined three classes of re-irradiated patients treated with IMRT by means of recursive partitioning analysis (RPA). RPA class I (>2 years after initial radiotherapy with resected tumors; 2 years overall survival: 62%) outperformed the class II (>2 years with unresected tumors or <2 years and without tracheostomy or feeding tube dependence; 2 years overall survival: 40%) and class III (remaining patients; 2 years overall survival: 17%) (79). Despite a potential selection bias due to the retrospective nature of the data, MIRI also demonstrated the redundancy of elective nodal irradiation and hyperfractionation regarding loco-regional control and overall survival. The same work indicated the need to administer ≥66 Gy equal dose in 2 Gy fractions to unresected tumors (80). This dose-tumor control relationship with conventional fractionation is also supported by the findings of a recent systematic review by the AAPM Working Group about hypofractionated SBRT, which shows superior tumor control with similar biologically 2 Gy/fraction equivalent doses of >35 Gy in 5 fractions, and suggests to administer 40–50 Gy in 5 fractions if possible (81).
  In another multi-institutional study, re-irradiation cohorts of IMRT and SBRT were compared using the same MIRI RPA classes II and III (no class I due to lack of operated patients). SBRT was associated with slightly less toxicity than IMRT (Grade ≥4 5.1% vs. 0.5%, *p* < 0.01). Both techniques showed similar overall survival in RPA class III, but significantly better survival with IMRT in class II. Comparable overall survival and loco-regional control were reported on RPA class II small tumors (≤ 25 cm^3^) with SBRT (>35 Gy in ≤ 5 fractions) and IMRT (77). After adjustment for potential confounders, SBRT and IMRT yielded similar overall survival and loco-regional control in the whole cohort. Either way, the patients seem to benefit from advanced technology by means of SBRT or IMRT compared to conventional techniques. Therefore, conservative reluctance to re-irradiation should be re-questioned. Validated tools for better patient selection criteria and prospective randomized studies to define the optimal strategies in re-irradiation setting are needed.
2. The Erasmus MC group published their results of T1–2 OPSCC cases treated with either pulsed-dose brachytherapy (*n* = 148; 22 Gy in 8 fractions over 24 h) or SBRT (*n* = 102; 16.5 Gy in 3 fractions over 1 week) boost following 46 Gy in 23 fractions with concomitant cisplatin (82). Toxicity and quality-of-life scores were comparable with both modalities. The authors favored the use of the non-invasive SBRT strategy, mainly based on the fact that it is less labor intensive, while brachytherapy is associated with perioperative and anesthesia-associated complications and requires specially trained personnel with hand dexterity.

## Conclusion

The findings of our survey indicate a low LOC among head and neck oncologists working in academic and multidisciplinary setting in 10 Swiss institutions. Regarding the results and the discussion concerning the specialties other than radiation oncology, the reader is advised to read the corresponding parts of this article. The highest LOC was achieved among medical oncologists, whereas the lowest was observed among head and neck surgeons. On the other hand, this level of disagreement may also depend on the topics chosen for the survey, and not necessarily the heterogeneity within the disciplines. It is also interesting to witness a low LOC regarding topics, where a high level of evidence actually does exist, and vice versa, such as definition of post-induction chemotherapy or post-operative treatment volumes, diagnostic modalities and time interval used to evaluate treatment response, use of boost techniques and dose/fractionation in early stage glottic laryngeal cancer. This article is expected to serve the head and neck oncologists to be aware of their discrepancies even among academic institutions and to stimulate discussion toward standardization of practice and prioritize topics of future clinical research. We support the concept of and the adherence to standardized guidelines, which should address controversial but relevant topics as well. Importantly, the level of evidence or the lack of thereof should always accompany the guideline recommendations. Last but not least, we would like to emphasize that this article series is not a literature review in the classical sense.

## Data Availability Statement

All datasets generated for this study are included in the manuscript/supplementary files.

## Author Contributions

GH, MB, OE, PD, and PP: conception and design. OE and PP: collection of data. All co-authors: generation of the initial and final versions of the questions, drafting of the manuscript and approval of the final version.

### Conflict of Interest statement

The authors declare that the research was conducted in the absence of any commercial or financial relationships that could be construed as a potential conflict of interest.
